# The Dwindling Microbiota of Aerobic Vaginitis, an Inflammatory State Enriched in Pathobionts with Limited TLR Stimulation

**DOI:** 10.3390/diagnostics10110879

**Published:** 2020-10-28

**Authors:** Eline F. M. Oerlemans, Sander Wuyts, Gert Bellen, Stijn Wittouck, Ilke De Boeck, Kateryna Ruban, Camille Nina Allonsius, Marianne F. L. van den Broek, Gilbert G. G. Donders, Sarah Lebeer

**Affiliations:** 1 Department of Bioscience Engineering, Research Group Environmental Ecology and Applied Microbiology, University of Antwerp, Groenenborgerlaan 171, B-2020 Antwerp, Belgium; eline.oerlemans@uantwerpen.be (E.F.M.O.); sander.wuyts@uantwerpen.be (S.W.); stijn.wittouck@uantwerpen.be (S.W.); ilke.deboeck@uantwerpen.be (I.D.B.); camille.allonsius@uantwerpen.be (C.N.A.); marianne.vandenbroek@uantwerpen.be (M.F.L.v.d.B.); 2 Femicare VZW, Clinical Research for Women, Gasthuismolenstraat 33, B-3300 Tienen, Belgium; gert.bellen@kuleuven.be (G.B.); katerina.ruban@femicare.net (K.R.); 3 Department of Obstetrics and Gynecology, Faculty of Medicine, University Hospital Antwerp, Wilrijkstraat 10, B-2650 Edegem, Belgium

**Keywords:** aerobic vaginitis, vaginal microbiome, next-generation sequencing, amplicon sequence variants, qPCR, dysbiosis, vaginal lactobacilli, bacterial vaginosis, Toll-like receptor 4, Toll-like receptor 2/6

## Abstract

While bacterial vaginosis (BV) is a well-known type of vaginal dysbiosis, aerobic vaginitis (AV) is an inflammatory condition that remains understudied and under-recognised. It predisposes women to serious complications including urogenital infections and pregnancy problems. Here, we investigated the bacterial community in AV to explore its possible role in AV pathogenesis. We collected vaginal lavage fluid samples of women (*n* = 58) classified by wet-mount microscopy as suffering from AV or BV and included an asymptomatic reference group without signs of AV or BV. AV samples showed reduced absolute abundances of bacteria in general and specifically of lactobacilli by qPCR, but *16S rRNA* gene sequencing and amplicon sequence variant analysis revealed that *Lactobacillus* remained the dominant taxon in 25% of the AV samples studied. The other AV samples showed high relative abundances of *Streptococcus agalactiae* and, unexpectedly, the anaerobes *Gardnerella vaginalis* and *Prevotella bivia* in more than half of the AV samples studied. Yet, despite increased relative abundance of these potential pathogens or pathobionts in the AV bacterial communities, the AV samples only slightly stimulated Toll-like receptor 4 and showed reduced activation of Toll-like receptor 2/6, receptors of two pathways central to mucosal immunity. Our findings indicate that the reduced total bacterial abundance with associated enrichment in certain pathobionts in AV might be mainly a consequence of the inflammatory conditions and/or altered hormonal regulation rather than bacteria being a major cause of the inflammation.

## 1. Introduction

Aerobic vaginitis (AV) is a highly relevant, yet still underexplored vaginal condition, first described by Donders et al. in 2002 [1]. Patients suffering from AV experience vaginal complaints such as abnormal discharge (pH 5.0–8.0 vs. normal pH 3.8–4.5), inflammation with redness and swelling, and small erosions or ulcerations. Following this, three main characteristics are at the basis of an AV diagnosis: (i) a variable amount of inflammation, (ii) thinning of the vaginal epithelium, and (iii) a disturbed bacterial community, deviating from the often-encountered high abundance of lactobacilli [2]. These three features are microscopically examined and numerically scored by evaluating the number and appearance of leukocytes, immature epithelial cells (or parabasal cells), as well as the presence of specific members of the bacterial community based on their morphologies. As these characteristics are also present in their most severe form in desquamative inflammatory vaginitis, this condition could also be seen as the most severe form of AV [1].

Symptomatic and asymptomatic forms of AV have been associated with pathologies that endanger general health and human reproduction, such as sexually transmitted infections including *Chlamydia* infection [3,4], progression of precancerous lesions of the cervix [5,6], severe vulvodynia causing introital pain and dyspareunia (i.e., experiencing pain during intercourse) [7], and numerous adverse outcomes of pregnancy [6,8,9,10]. Regardless of the associations with such serious health problems, research regarding abnormal vaginal bacterial communities has up to now mainly focused on bacterial vaginosis (BV). BV can be recognized by (i) a malodorous vaginal discharge, (ii) an elevated pH of 4.5–5.5, and (iii) a healthy, non-inflamed vaginal epithelium covered with a biofilm that can be observed using microscopy and consists of *Gardnerella vaginalis* and other species [11]. Regarding the microbial community composition, studies indicate that the number of lactobacilli is reduced in BV [12,13,14], while anaerobic species such as *G. vaginalis*, *Atopobium vaginae*, *Prevotella*, *Mobiluncus*, and *Dialister* species overgrow the niche (as previously reviewed in Van De Wijgert et al., 2014 and Petrova et al. 2015 [2,14]).

Selective bacterial culturing, microscopy data, and quantitative PCR (qPCR) have described features of the bacterial community in AV. Besides the abovementioned depletion of *Lactobacillus* species, based on classical culture techniques, it was suggested that aerobic cocci or small bacilli such as group B streptococci, *Staphylococcus aureus*, *Escherichia coli*, and enterococci are (slightly) increased in the vaginal microbiome of AV patients [1,15,16], hence the term “aerobic vaginitis”. Next-generation sequencing (NGS) studies exploring the AV microbial community are currently lagging behind, although they could provide insights at a community-broad level, and identify members up to the subgenus level, without the possibility of culture bias by fast-growing aerobic bacteria.

AV shares some of the hallmarks of BV, such as a malodorous vaginal discharge and an elevated vaginal pH. Because of this and since BV is better known, AV is sometimes misdiagnosed as BV. Wet-mount microscopy can easily distinguish these conditions, but microscopy combined with adequate scoring systems remains largely underused in clinical practice [9]. Such a microscopic approach is easily mastered with an intense short course of training and a relatively simple phase-contrast microscope with ×400 magnification is sufficient for the analysis [17]. However, when this approach is not applied, women with serious symptoms and/or health risks could be left without proper treatment. Since AV and BV differ (e.g., in their inflammatory profile), different factors might lie at the basis of these conditions, so different therapies might be necessary to treat them. For both AV and BV, the underlying causes have not yet been elucidated. For AV, a dysregulation of the immune system, a lack of oestrogen, lichen planus (i.e., dermatological condition caused by an inflammatory reaction of skin and/or mucosa), and vitamin D deficiency have been suggested as causative or contributing factors, but these need to be substantiated further [15].

Here, we hypothesized that an integrated molecular approach could provide additional information regarding the important members of the bacterial community in AV and improve our understanding of AV pathogenesis. Following this, the primary goal of our study was to provide a detailed, community-wide DNA-based insight into the vaginal microbiome of AV patients. We used high-throughput *16S rRNA* gene sequencing and processed the samples at a single nucleotide level, allowing us to discriminate closely related biological variants, termed amplicon sequence variants (ASVs) [18]. Because NGS alone lacks the ability to provide absolute abundance, it was important to combine NGS with more quantitative methods such as quantitative PCR (qPCR). Furthermore, to explore the microbial communities as the stimulus preceding the observed inflammation in AV, we investigated whether the identified bacteria could also activate Toll-like receptor 4 (TLR4) and the dimer of TLR2 and TLR6. TLRs form a vital part of host-responses at mucosal sites as they are the initiators of important and sensitive pathways of the innate immune system, which can lead to activation of NF-κB (nuclear factor kappa-light-chain-enhancer of activated B cells) and immunostimulatory signaling [19], relevant in light of the pro-inflammatory nature of AV. In fact, an important pathway of how Gram-negative bacteria (especially *Enterobacteriaceae*) can trigger inflammation is through activation of TLR4, the bacterial-responsive TLR that induces most often and most vigorously a pro-inflammatory response [20]. The dimer of TLR 2 and TLR6 is responsive to a range of bacterial structures, including peptidoglycan, lipopeptides, atypical LPS (such as from *Prevotella* species), and lipoteichoic acid. The latter is present in the cell wall of Gram-positive bacteria, including lactobacilli and group B *Streptococcus* [21,22,23,24,25]. Here, we tested the potential of (a subset of) the samples to stimulate TLR4 and TLR2/6 in NF-kB-reporter cell lines. This combination of *16S rRNA* sequencing, qPCR, and TLR assays brought some specific features of AV to light and allowed us to discriminate AV, BV, and reference samples. Our findings therefore did not only increase our understanding of AV, but are also relevant for diagnostics.

## 2. Materials and Methods

### 2.1. Study Population and Ethical Approval

58 premenopausal women between 18 and 51 years of age showing a normal *Lactobacillus*-dominated microbiota (*n* = 18) or suffering from BV (*n* = 20) or AV (*n* = 20) were recruited at the general hospital Heilig Hart, Tienen, Belgium. The study was reviewed and approved by the ethical committee of the Heilig Hart hospital in Tienen (Nr 20040719, 19th of July 2004) and all patients gave their explicit consent before sampling.

### 2.2. Sample Collection and Diagnosis

Wet-mount microscopy was used for diagnosis, as previously described [1]. The images were scored for lactobacillary grades, BV score (similarly to Nugent scoring), AV score (proportional number of leukocytes, appearance of leukocytes, and presence of parabasal cells and small bacilli and cocci) and presence of *Candida*. The group where the wet-mount microscopy showed a lactobacillary grade I or IIa, without signs of AV, BV, or *Candida* [1], acted as a reference group. For DNA extraction and cell experiments, vaginal lavage fluid was collected by flushing and reaspirating 3 mL of sterile saline through a 0.5 mm wide and 6 cm long needle in the left, central, and right upper vaginal vaults, as previously described [1]. The fluid was subsequently frozen at −80 °C at Femicare Clinical Research Center (Tienen) until later analysis. Samples were transported on dry ice to the lab and processed immediately after thawing. DNA was extracted from 700 µL of the lavage fluid by means of the PowerFecal DNA isolation kit (Qiagen, Hilden, Germany), according to manufacturer’s instructions. As a control for contamination originating from the kit buffers, a blank extraction was included whenever opening and finishing the kit.

### 2.3. 16S rRNA Gene Sequencing with Miseq and qPCR

For every DNA sample, two PCR reactions amplifying the V4 region of the *16S rRNA* gene were performed as two technical repeats. The primer design and cycling conditions were based on the paper of Kozich et al. (2013) [26] (Table S3). The PCR-products were purified using Agencourt AMPure XP magnetic beads (Beckmann Coulter, Brea, CA, USA), according to manufacturer’s instructions, and eluted in PCR-grade water. DNA concentrations were estimated with Take3 micro-volume plate (BioTek, Winooski, VT, USA) for equimolar pooling. The resulting amplicon library was loaded onto a 0.8% agarose gel for gel purification of the amplicon band (Nucleospin gel and PCR clean-up kit; Macherey-Nagel, Dueren, Germany). The purified library was quantified by Qubit (Thermo Fisher, Waltham, MA, USA) and diluted to 2 nM. The library was loaded onto the flow cell of a v2 500 cycle MiSeq kit (Illumina, San Diego, CA, USA) at 7 pM and spiked with 10% PhiX DNA. MiSeq sequencing was performed at the Antwerp University Hospital, Belgium (MiSeq M00984).

### 2.4. Bio-Informatics

Sequencing data were analyzed using the DADA2 package [18] in the R environment following the DADA2 standard operating procedure. In summary, sequences with at least one ambiguous base, reads containing the lowest possible quality score 2, and reads that have more than two “expected errors” were discarded. After visual inspection of the read quality, the first 12 base pairs were trimmed and the forward reads were truncated at position 240 while the reverse reads were truncated at position 220. Finally, DADA2′s core algorithm was performed, followed by read merging. Taxonomy was assigned using Silva v123 [27] and further manual classification was done using EZtaxon (Chunlab, Korea) [28]. The sequence table and taxonomy were then imported into the Phyloseq package [29] and further processed. At this point, sequences of PCR technical replicates were pooled, resulting in one merged sample. The compositional analysis of differential abundance for pairwise comparison between the AV, BV, and normal *Lactobacillus*-dominated (NL) groups was based on the ANCOM method [30]. Read counts were first aggregated on the genus or ASV level. For each combination of a target genus/ASV and a reference genus/ASV, the log ratio of their relative abundances was calculated for each sample. Pseudocounts of one were first added to avoid division by zero. Subsequently, differential abundance between two groups was assessed by a Wilcoxon rank-sum test per target/reference taxon combination. Significant differences were determined from the Wilcoxon *p*-values by capping the false discovery rate at 10% with the method of Benjamini and Yekutieli [31]. Quantification of differential abundances was performed using the two-sample Hodges–Lehmann estimator (the median of all pairwise differences between the samples).

### 2.5. qPCR

The previously isolated DNA of the samples was diluted 20 fold in qPCR grade water (Invitrogen, Carlsbad, CA, USA) and used to determine bacterial and human cell concentrations by qPCR, using the StepOnePlus real time qPCR system (Applied Biosystems, Foster City, CA, USA) and SYBR^®^ Green chemistry (PowerUp™ SYBR^®^ Green Master Mix, Applied Biosystems, Foster City, CA, USA). DNA was extracted from 500 µL and 100 µL of overnight cultures of *Lactobacillus crispatus* LMG12005 and *Streptococcus agalactiae* ATCC49447 and from 500 µL and 75 µL of a culture of THP-1 monocytes (kindly donated by professor Peter Delputte, LMPH, University of Antwerp), using the same protocol as for the samples (Table S2). The obtained DNA was serially diluted (10 fold dilutions) and used as specific standard curve. Further, 5 µL of DNA (sample or standard) was used for every 20µL reaction, performed in duplicate. Primer sequences can be found in Table S3.

### 2.6. HEK-Blue™ hTLR4 and hTLR2/6 Experiment

The ability of the samples to stimulate TLR4 and the dimer of TLR2 and TLR6 was estimated through the use of the HEK-Blue™ hTLR4 and the HEK-Blue™ hTLR2-TLR6 reporter cell line (Invivogen, San Diego, CA, USA; the former kindly donated by proffesor Rudi Beyaert), which produces secreted embryonic alkaline phosphatase in response to TLR4 or TLR2-TLR6 stimulation. The day prior to an experiment, cells were seeded in a 96 well plate at a concentration of 2.5 × 10^5^ cells/well (details on growth conditions can be found in Table S2). Samples were thawed and diluted 1:10 with fresh growth medium (DMEM with 10%fetal calf serum, Gibco, Carlsbad, CA, USA) and added to the wells (100 µL/well) in triplicate. A lipopolysaccharide (LPS) standard, isolated and purified from *E. coli* K12 (LPS-EK, Invitrogen, San Diego, CA, USA), was diluted to a final concentration of 10 ng/mL and used for the HEK-Blue™ hTLR4 cells. PAM2CSK4 was resuspended and diluted to a final concentration of 50 ng/mL and used as a positive control for the HEK-Blue™ hTLR2-TLR6 cells. The cells were incubated with the standards/samples for 24 h at 37 °C, 5% CO_2_. The embryonic alkaline phosphatase secreted by the cells was quantified by adding 50 μL of supernatant of each well (in duplicate) to 100 μL of substrate solution (1.5 mg/mL pNPP, 150 mM Tris-HCl, 150 mM NaCl, and 7.5 mM MgCl_2_ at pH 9.5). After 20 min of incubation shielded from light, absorbance was measured at 405 nm.

### 2.7. Statistical Analysis

All statistics and visualizations were performed using RStudio (v. 1.1.383, Boston, MA, USA), ggplot2, and ggpubr packages. For pairwise comparisons between the groups (qPCR and TLR reporter cell experiments), pairwise Wilcoxon tests were used, corrected for multiple testing with the Holm-Bonferroni method.

### 2.8. Availability of Data

The complete sequencing analysis pipeline is available at https://github.com/swuyts/AV_BV_study and the data can be accessed through ENA accession number PRJEB29686. qPCR data is available as Supplementary dataset 1.

## 3. Results

### 3.1. Taxonomic Profiles of AV Bacterial Communities

**Figure 1:**
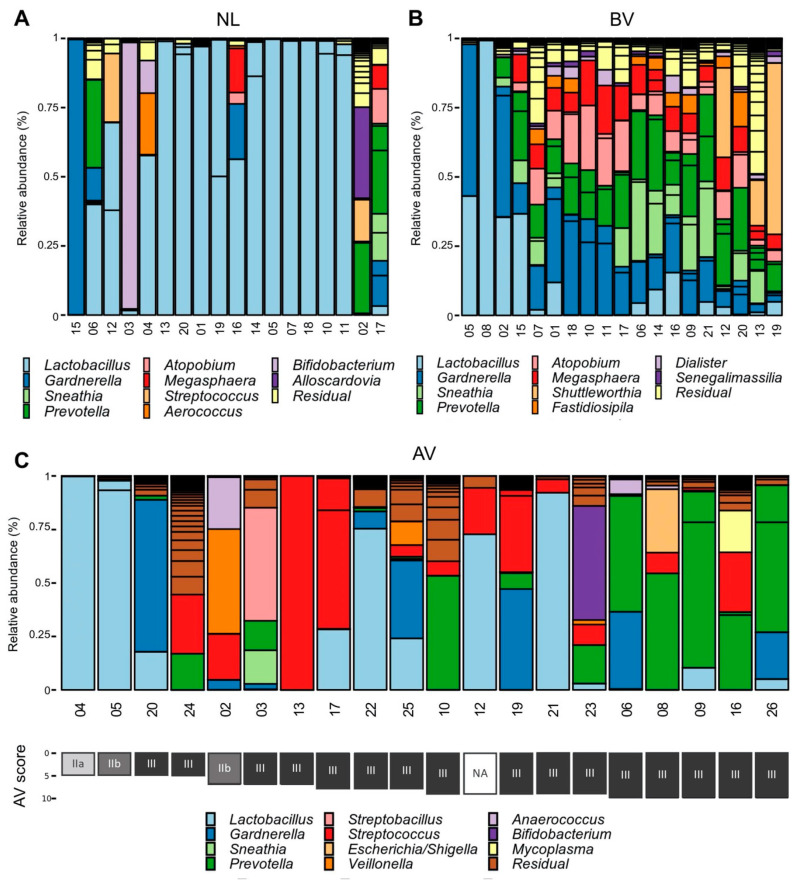
Bar graphs showing the taxonomic composition of the samples. Samples are categorized as (**A**) reference sample (NL), (**B**) BV, and (**C**) AV based on wet-mount microscopy (1). The abundances of the 11 most abundant genera are indicated in the bar graph with the *y*-axis corresponding to the relative abundance. Bars are colored according to genus or as residual in case the amplicon sequence variant (ASV) did not belong to the 11 most abundant genera. Samples are ordered according to similarity by minimizing Bray-Curtis distance between neighboring samples (**A**,**B**) or AV score (**C**). AV score is indicated below the sample number, colored according to lactobacillary grade (one component of composite AV score). AV: aerobic vaginitis, BV: bacterial vaginosis, NL: reference group with a lactobacillary microbiota (Lactobacillary grade I, no signs of AV or BV) according to microscopy.

The vaginal bacterial communities of women suffering from AV (*n* = 20) or BV (*n* = 20, 19 samples passed quality control), diagnosed by conventional phase contrast microscopy (Table S1 and Figure S1), and a reference group of women (*n* = 18) with a normal *Lactobacillus*-dominated (NL) microscopy image were characterized by *16S rRNA* gene sequencing. Although the classification in NL, BV, and AV of our samples was initially based on wet-mount microscopy (and thus, mainly based on bacterial morphotypes, inflammatory cells, and quality of host cells) [1], *16S rRNA* gene sequencing analysis confirmed that most samples of the NL and BV group showed distinct community compositions as expected (Figure 1, Figure S2). Half of the *Lactobacillus*-dominated group (NL) was almost exclusively dominated by *Lactobacillus* sequences (>95% relative abundance) (Figure 1) and in five other samples (NL04, NL06, NL12, NL16, and NL19), high relative abundances of *Lactobacillus* species (31.9–58.0%) were observed. In four samples (NL02, NL03, NL15, NL17), no or only few lactobacilli could be detected (<5%), but this is in agreement with previous work that not all healthy women are dominated by lactobacilli [14]. In the BV classified group, almost all samples were characterized by a typical BV microbiome profile with co-occurrence of *Atopobium*, *Gardnerella*, *Prevotella*, *Dialister*, *Sneathia*, and *Megasphaera* as the most important genera.

The microbiome profiles of the AV group were characterized by dominance of only a few ASVs in every sample (Figure 1). The four genera most prevalent in the AV group (only taking ASVs into account when they exceeded 1% relative abundance) were *Lactobacillus* (dominant with a relative abundance above 50% in 5/20, present in 11/20 samples), *Prevotella* (dominant in 5/20, present in 13/20 samples), *Streptococcus* (dominant in 2/20, present in 13/20 samples), and *Gardnerella* (dominant in 1/20, present in 8/20 samples). The occurrence of *Prevotella* was associated with higher AV scores (Pearson’s correlation *r* = 0.6, *p* = 0.008, also when lactobacillary grades were not taken into account for the AV score, *r* = 0.57, *p* = 0.01, Supplementary Figure S3), while the relative abundances of *Lactobacillus, Streptococcus*, and *Gardnerella* were not correlated with AV scores. Various other genera were also detected, including *Veillonella*, *Dialister*, *Anaerococcus*, *Streptobacillus*, *Sneathia*, and *Escherichia/Shigella*, but these were only present in low relative abundances and/or occurred only in one or a few samples.

The dominance of lactobacilli in AV may seem unexpected because a higher lactobacillary grade (which corresponds to other bacteria gaining dominance over lactobacilli) is one of the three main features of microscopic AV diagnosis. Nonetheless, lactobacillary grades are just one element in the composite score of AV, explaining the high relative abundance of lactobacilli in some samples (here, >50% in 5/20). Upon compositional analysis of differential abundance (using our own implementation of ANCOM analysis [30]), we found the strongest differences in abundance between the AV and NL group for the genera *Lactobacillus* (more abundant in the NL group), *Prevotella*, and *Streptococcus* (more abundant in AV) (Figure S4a). Also, *Dialister* and *Anaerococcus* showed to be overrepresented in the AV group (Figure S4a). *Dialister* and *Anaerococcus* were present (>1% relative abundance) in seven samples but dominant (>50% relative abundance) in none. These differences did not reach statistical significance for AV versus NL, possibly due to a lack of statistical power (low sample size). However, when comparing AV versus BV, many genera were found to be significantly differentially abundant, with the strongest effects for *Streptoccocus* (more abundant in AV), *Megasphaera, Atopobium*, and *Sneathia* (more abundant in BV; Figure S4b).

**Figure 2:**
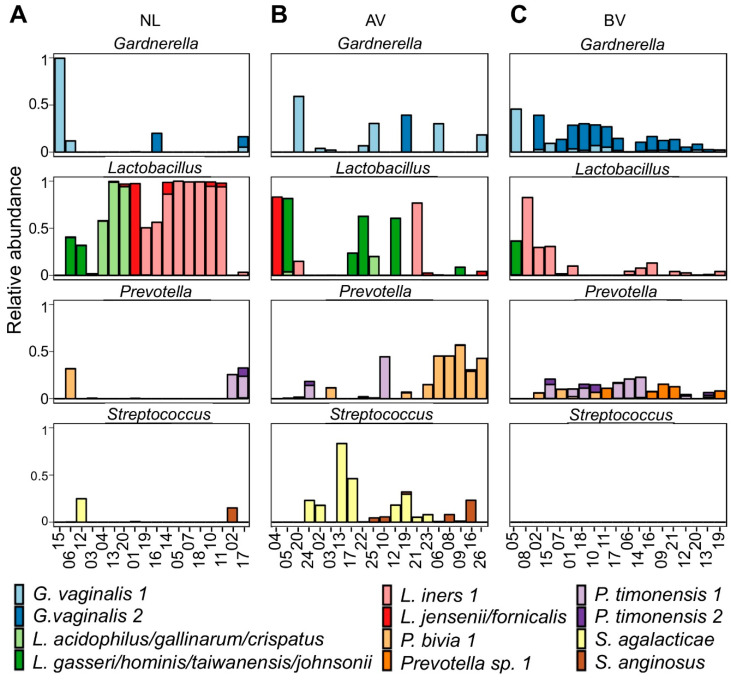
Relative abundances of *Lactobacillus, Gardnerella*, *Prevotella*, and *Streptococcus* ASVs in the NL (**A**), BV (**B**), and AV group (**C**). Bars are colored according to ASV. Only ASVs that accounted for at least 5% of sequences in at least two samples are visualized. ASVs were classified by EZBioCloud (27) as the closest matching species (allowing for a maximum of two mismatches). AV: aerobic vaginitis, BV: Bacterial Vaginosis, NL: reference group with a normal lactobacillary microbiota (Lactobacillary grade I-IIa, no signs of AV or BV) according to microscopy.

As *Lactobacillus, Prevotella, Streptococcus*, and *Gardnerella* constituted the most abundant genera across the entire dataset in terms of relative abundance, the most abundant ASVs of these genera were classified further by EZ BioCloud [28] to a sub-genus level based on single nucleotide resolution when possible (Figure 2). In total, 17 *Lactobacillus* ASVs were found in the dataset. The four most abundant *Lactobacillus* ASVs were classified as *L. iners, L. crispatus/acidophilus/gallinarum, L. gasseri/hominis/taiwanensis/johnsonii*, and *L. jensenii/fornicalis*, in agreement with community groups III, I, II, and V proposed by Ravel et al. (2011), respectively. Interestingly, although *L. iners* was the most abundant ASV in BV and our reference group, followed by *L. crispatus/acidophilus/gallinarum*, this was not the case for the AV group, where three of the five subjects were still dominated by *L. gasseri* (or related). In total, 26 different *Prevotella* ASVs were found in the dataset. The most abundant *Prevotella* ASV was classified as *Prevotella bivia*, while two others were classified as *Prevotella timonensis* and a fourth abundant *Prevotella* ASV could not be classified further than to genus-level. The dataset contained six *Streptococcus* ASVs and two of them occurred in multiple samples at higher relative abundances (up to 100% and 27.9% relative abundance), which could be identified as *Streptococcus agalactiae* (i.e., group B *Streptococcus*) and *Streptococcus anginosus*, respectively. The *Streptococcus* ASVs were limited to the AV group, except for two NL samples, NL12 and NL02 (at 15.1% and 24.8% relative abundance, respectively). Our dataset also contained four *Gardnerella* ASVs of which two occurred often (in 24/58 and 17/58 samples) and in higher relative abundances (up to 99.6% and 47.2%). Both sequences were found in BV, AV, and the reference group and were both derived from *Gardnerella vaginalis* strains. These ASVs matched perfectly with the sequences of *G. vaginalis* variants reported by Callahan et al., 2017 [32], including their variant which was solely associated with preterm birth (cfr. our *G. vaginalis* ASV1) (Figure 2).

When we looked into the compositional analysis of differential abundance of the ASVs in the AV versus the NL group, we found *Lactobacillus iners* 1 was significantly underrepresented in the AV group (Supplementary Figure S4c). On the other hand, *Prevotella bivia* 1, *Streptococcus agalactiae* and *Streptococcus anginosus*, and one *Dialister* ASV (identified as *Dialister propionicifaciens*) were overrepresented in the AV group as compared to the NL group. However, the latter differences were not statistically significant, indicating that the mere bacterial composition of the vaginal microbiota can be insufficient to discriminate AV from NL. On the other hand, similar to the genus-level analysis, multiple significant differences in abundance were found for AV and BV, such as for ASVs belonging to the genera mentioned before (i.e., *Streptococcus, Megasphaera, Atopobium*, and *Sneathia*; Supplementary Figure S4d). However, *Gardnerella vaginalis* 2 also showed to be highly differentially abundant, being much more abundant in BV as compared to AV.

### 3.2. Bacterial Counts of the Entire Community and Specific Community Members

As *16S rRNA* gene sequencing can only give semi-quantitative results, and did not reveal a lot of differences between AV and NL, we complemented these data with qPCR, allowing us to estimate and compare absolute numbers of human and bacterial cells in the samples, as well as selectively of lactobacilli, streptococci, *Enterobacteriaceae*, and staphylococci, i.e., taxa which have previously been proposed for diagnosis of AV [16].

**Figure 3:**
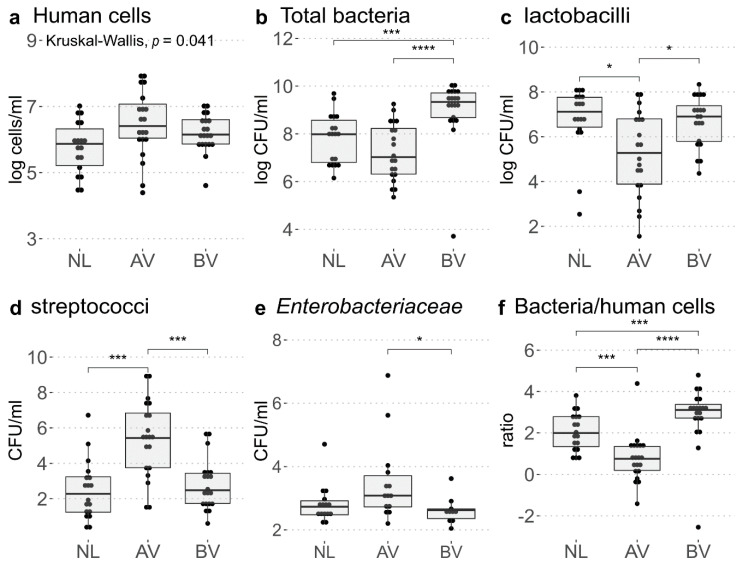
Estimated human cell (**a**) and bacterial concentrations (**b**–**d**); (**a**) Concentration of human cells, estimated by qPCR of the *cyc1* gene. A Kruskal-Wallis test indicated significant differences between the groups (*p* = 0.041), but this was not confirmed by pairwise Wilcoxon tests. (**b**) Total bacterial concentration estimated by qPCR with general primers for the V4 region of the *16S rRNA* gene. (**c**) Concentration of lactobacilli, (**d**) streptococci, and (**e**) *Enterobacteriaceae*, estimated by qPCR with genus-specific primers. (**f**) Ratio of bacteria to human cells as an estimator for bacterial load. For each gene, a standard curve was used to calculate CFUs or cell concentrations, as indicated in Materials and Methods and Supplementary Figures. The significance levels from Wilcoxon and Kruskal-Wallis tests indicated in the graphs correspond to ns: *p* > 0.05, *: *p* < 0.05, ***: *p* < 0.001, ****: *p* < 0.0001.

The highest concentrations of human cells, estimated by qPCR of the *cytochrome c1* gene, were found in the AV group (median = 2.7.10^6^ cells/mL), followed by the BV group (median = 1.4 × 10^6^ cells/mL) and then the reference group (median = 7.4 × 10^5^ cells/mL) (Figure 3a). Higher eukaryotic DNA loads are expected in the AV group due to large amounts of parabasal cells and/or leukocytes typically seen in AV lavage fluids, resulting from the epithelial thinning and inflammation, respectively [1]. Larger differences between the groups were observed for total bacterial concentrations (Figure 3b). The BV group showed the highest concentrations (median = 2.2 × 10^9^ CFU/mL) and was significantly higher than those of the reference group (median = 9.6 × 10^7^ CFU/mL, *p* = 0.00034) and the AV group (median = 1.1 × 10^7^ CFU/mL, *p* = 6 × 10^−6^). Looking at specific genera with genus-specific primer sets, the reference group had the highest concentration of lactobacilli (median = 1.5 × 10^7^ CFU/mL AV-NL *p* = 0.021; Figure 3c) and lowest concentration of streptococci (median = 254 CFU/mL, AV-NL *p* = 0.0005; Figure 3d), while this was reversed for the AV group (median concentration of lactobacilli = 2.2 × 10^5^ CFU/mL, AV-BV *p* = 0.024, and streptococci = 2.7 × 10^5^ CFU/mL, AV-BV *p* = 0.0008). Even though the relative abundances were lower, the BV group had somewhat similar concentrations as the reference group for both lactobacilli (median 8.2 × 10^6^ CFU/mL) and streptococci (median = 3.10^2^ CFU/mL). In contrast to the streptococci, the association of AV with two other bacterial groups, *Enterobacteriaceae* and *Staphylococcus*, was less evident from our data. For *Enterobacteriaceae*, 14 of 20 AV samples showed amplification (Ct > 38; median = 1.2 × 10^3^ CFU/mL), with three samples showing estimated concentrations above 10^4^ CFU/mL (Figure 3e). This was statistically higher than the BV samples (amplification in 9/19 samples with a median of 4.2 × 10^2^ CFU/mL), but not the reference samples (amplification in 10/18 samples with a median of 5.4 × 10^2^ CFU/mL). For staphylococci, we found that several samples also did not amplify *Staphylococcus* DNA (Ct > 38), (3/18, 6/20, and 6/19 in the reference group, AV group, and BV group, respectively). However, in the other samples, we observed relatively similar concentrations of staphylococci in the three groups (Supplementary Figure S5; median AV = 2.3 × 10^5^ CFU/mL, median NL = 1.5 × 10^5^ CFU/mL, median BV = 3.2 × 10^5^ CFU/mL). Nevertheless, despite their similar DNA abundance, their activity could be altered between the two states (AV and reference) because of the different abundance of lactobacilli. For example, we tested six vaginal *Lactobacillus* isolates of various species and their secreted molecules in spent supernatant were all able to inhibit the growth of *S. aureus*, in contrast to *Streptococcus salivarius* and *E. faecium* (Figure S6). This can indicate that since vaginal lactobacilli and staphylococci often co-occur and therefore compete for resources, these organisms have developed strategies to inhibit each other’s growth.

When comparing the total bacterial load as a ratio of the concentration of total bacteria to the concentration of human cells, the three groups could be clearly distinguished (Figure 3f). The highest values were found for BV (median = 1282, AV-BV *p* = 1.7 × 10^−5^ and BV-NL *p* = 0.004), intermediate values for NL (median = 99), and the lowest values for AV (median AV = 5.7, AV-NL *p* = 0.0003).

### 3.3. Toll-like Receptor Activation Capacity

**Figure 4:**
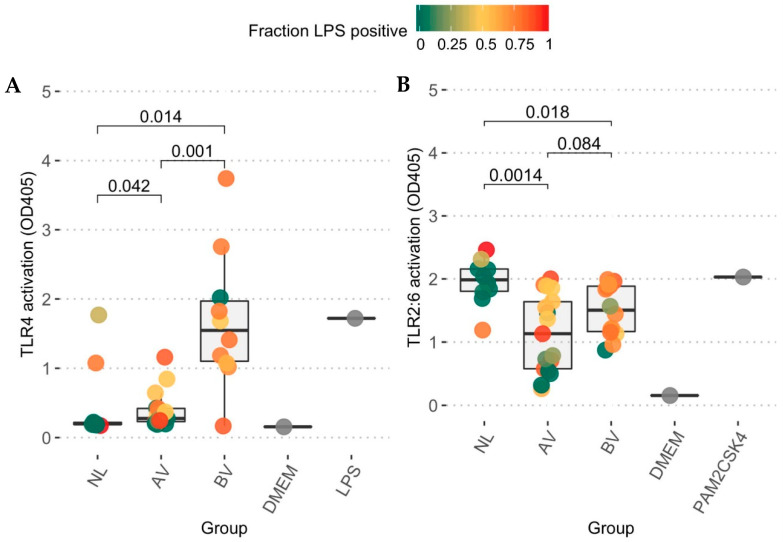
HEK-Blue hTLR4 and HEK-Blue hTLR2-TLR6 cell response after stimulation of diluted samples. Formation of p-nitrophenol, generated by alkaline phosphate, induced by (**A**) TLR4 or TLR2-TLR6 (**B**) stimulation was measured through spectrophotometry by its absorbance at 405 nm (*Y*-axis). The individual dots are colored according to the cumulated relative abundances of LPS positive taxa (Gram-negative and Gram-variable) as LPS is the clear ligand of TLR4, while the dimer of TLR2 and TLR6 is generally more responsive to Gram-positive ligands (peptidoglycan, lipoteichoic acid). The grey dots indicate the blank measurements (mean = 0.156 and 0.155) and the positive controls (LPS, 10 ng/mL, mean = 1.72 ± 0.05 sd and PAM2CSK4 50 ng/mL, mean = 2.03 ± 0.07 sd). *p*-values are indicated, as obtained from a pairwise Wilcoxon test corrected for multiple testing.

After characterization of bacterial abundance and taxonomic composition of the AV microbiome, we subsequently monitored the ability of the microbially characterized vaginal lavage samples to stimulate the innate immune system through TLRs. Limited activation of TLR4 was observed in most samples of the AV group, which yielded only slightly higher activation (*p* = 0.04, pairwise Wilcoxon test) as compared to the reference group (Figure 4A). In the BV group, nine out of 10 tested samples were able to stimulate TLR4, yielding a significantly higher response than the AV group (*p* = 0.001) and the reference group (*p* = 0.023). In contrast, in the TLR2-TLR6 assay (Figure 4B), the *Lactobacillus*-dominated reference group showed the highest stimulation (median = 1.99), followed by the BV group (median = 1.50, BV-NL *p* = 0.02) and finally the AV group (median = 1.13; AV-NL *p* = 0.001). However, we acknowledge that while such minimalistic models are useful to eliminate other signals and focus just on particular immune pathways, this approach is too limited to generalize to a possible inflammatory response of the host to the resident bacteria since many other pathways could be involved as well.

## 4. Discussion

Vaginal dysbiosis affects millions of women annually, having a significant impact on their health and fertility [33]. Here, we studied the microbial community in AV with molecular techniques, which revealed a markedly reduced bacterial load in AV and the presence of group B *Streptococcus* and *Prevotella bivia* as important discriminatory vaginal community members. Moreover, we could show altered stimulatory potential for TLR4 and TLR2-TLR6 of AV samples. These features can not only increase our understanding of AV as a concept, but could also be applied in future diagnostic tools as here we could discriminate AV, BV, and reference samples from each other.

Studies using *16S rRNA* sequencing had not yet been applied for AV. Previous studies based on culture, microscopy, and qPCR indicated an increased presence of aerobes such as group B streptococci, *S. aureus, E. coli*, and enterococci and depletion of lactobacilli in AV [1,16]. Here, we did find high relative and absolute abundances of *Streptococcus* ASVs in two thirds of the AV samples (13/20 > 1% relative abundance and 14/20 > 10^4^ CFU/mL). Therefore, our results support the previous suggestions of an increased prevalence of streptococci in AV (cfr. culture and qPCR [1,16]). This is highly relevant with group B *Streptococcus* being a leading cause of serious neonatal infections [34] and is thus of value for early stratification of women at risk for this complication. For *Enterobacteriaceae* and *Staphylococcus*, our *16S rRNA* gene deep sequencing approach did not show (multiple) ASVs with high relative abundance and our qPCR analysis did not indicate elevated concentrations. Nonetheless, we could have underestimated the role of staphylococci since it is plausible that the activity of the staphylococci is different in AV due to the lack of competing microbes such as lactobacilli with bacteriostatic effects on *S. aureus* growth. Although some AV samples did show *Lactobacillus* as a dominant taxon in the *16S rRNA* analysis, overall, *Lactobacillus* (and especially *Lactobacillus iners* 1) showed a lower abundance in the AV group as compared to the NL samples in the compositional analysis. Additionally, in accordance with previous studies, the AV samples tested here also exhibited lower absolute numbers of lactobacilli in qPCR analyses. This could suggest that although lactobacilli are diminished in numbers, this did not result from other bacteria becoming more abundant and outcompeting them, but rather from host (immune) factors killing or inhibiting their growth (e.g., by reducing sugar availability).

In contrast to previous culture-based studies and what the name ‘aerobic” vaginitis would suggest, we also unexpectedly recovered the anaerobic *Prevotella*, *Gardnerella*, and *Dialister* from the AV samples. These genera were not previously found in AV and were only associated with BV (or community state type IV) up to now [12,14]. This suggests that although AV and BV are very different conditions with different clinical presentations and bacterial communities, they also share some features in their specific microbiota. However, metronidazole, the standard treatment for BV, is not effective in treating AV [1], but will affect the presence of *Gardnerella*, *Prevotella*, and *Dialister* [35,36]. As the clinical symptoms they would cause (inflammation-grade/epithelial shedding) are also so different in AV compared to BV, it is unlikely that these taxa are solely responsible for causing AV. Nonetheless, these could probably still contribute to symptoms or complications. In fact, *Prevotella bivia* (found in 9/20, predominantly severe, AV samples) is known to produce enzymes such as sialidases that might contribute to mucosal degradation and thus epithelial thinning in AV [37,38]. Furthermore, increased sialidase concentrations have been found previously in AV [10] and both sialidase activity and *P. bivia* have been linked to preterm delivery [39]. Therefore, finding commonalities in the bacterial communities of AV and BV still provides an interesting route to explore the associated health complications that AV and BV share, such as preterm birth [9,32,40,41].

For the assay evaluating TLR2-TLR6 responses, induction was most remarkable in the reference group. This could possibly be explained by a large presence of lactobacilli in these samples [23]. Importantly, while traditionally TLR activation is linked with pro-inflammatory responses, stimulation of TLR2-TLR6 has been proposed to also have an anti-inflammatory role in certain circumstances [19]. As the vaginal epithelium is proposed to be in a controlled state of inflammation [21], this immunomodulatory role of TLR2-TLR6 activation by the resident *Lactobacillus*-dominated commensal community might be an important part of maintaining homeostasis in the reference group. Furthermore, the lower activation of TLR2-TLR6 observed in the AV samples as compared to reference samples, and possibly, therefore, lower anti-inflammatory activity, combined with the slight activation of pro-inflammatory TLR4 signalling might affect this delicate immunological balance in the vaginal mucosa. This lack of clarity highlights the importance to study the interactions between the immune system and the AV microbial community.

The possibility of immune dysfunction itself as an underlying cause of AV has already been the subject of previous research [4]. While we could not find strong clues of inflammation triggered by TLR4 or TLR2-TLR6 in AV, there are many other bacterial factors that could cause inflammation via other pathways. However, if AV were the result of a disturbed immune response, the present microbial community would not trigger the disease but rather be shaped by it, selecting for bacteria with immune evasion strategies such as group B *Streptococcus* and *P. bivia* [25,42], while the activity of *S. aureus* might shift to a more virulent phenotype because of the absence of lactobacilli [43]. Several of our observations favour the hypothesis of an underlying host-derived cause in AV: (i) a clear heterogeneity in the microbial community, (ii) presence of all four “typical” lactobacilli, (iii) a decreased abundance of all bacteria, including lactobacilli, and finally, (iv) a limited ability to stimulate TLR4 and TLR2-TLR6. These findings could also be explained by a second interesting hypothesis concerning the causation of AV, which suggests that the bacterial community is disturbed by impaired function of oestrogen receptors [4]. This would lead to a reduction of the superficial cells of the vaginal wall, with a decrease in glycogen availability and subsequently, lactobacilli as a result. This local disturbance could then pave the way for other bacteria to increase in abundance by, for example, loss of lactate production and bacteriocins, but not to the concentrations seen in BV due to the limited availability of carbon sources.

Here, we describe a first explorative study of the AV microbiome using NGS, however currently our main shortcoming is our sample size. Therefore, our findings should be confirmed in larger and more diverse cohorts. If such studies confirm the hypothesis that the altered microbial community is the result of the disease rather than the cause of it, this would hold important implications regarding the treatment options and AV as a concept. For example, it would argue against the use of antibiotics for the treatment of AV [44] as they would just temporarily clear the remaining bacterial community disturbed by the inflammation or epithelial thinning. At the moment, antibiotics have been suggested as one of three cornerstones of AV treatment, but based on the above mentioned hypotheses (immune dysfunction or impaired receptor function), two other proposed main therapeutic strategies, topical steroids and oestrogens, are likely to be more effective in the long term [15]. Lastly, these findings also have important implications for the development of probiotics targeting AV microbiota and *Lactobacillus* restoration since the inflammation and associated antimicrobial environment will complicate the survival and efficacy of the probiotics in these patients. Selection strategies will therefore benefit from in vitro prediction models taking this into account [45].

## Supplementary Materials

The following are available online at https://www.mdpi.com/2075-4418/10/11/879/s1, Supplementary dataset S1: raw data of qPCR and HEK-cell experiments., Table S1: features for microscopic scoring of wet-mounts (1). Table S2: Biological materials (DNA, cell lines, and bacterial cultures) used in qPCR and HEK cell experiments. Table S3: Primer sequences used for 16s rRNA sequencing and qPCR. Figure S1. Classification of groups based on microscopy. Figure S2: PCOA plot indicating beta-diversity at ASV level (A) and genus level (B) between all samples of AV, BV, and reference (NL) groups. Figure S3: Correlations between AV-scores and relative abundances bacterial taxa. Figure S4: Compositional analysis of differential abundance. Figure S5: Supplementary information qPCR results. Figure S6: Bacteriostatic effect of spent culture supernatant of vaginal lactobacilli on *S. aureus*. References supplementary tables and figures.
