# Supplementary material for: The Dwindling Microbiota of Aerobic Vaginitis, an Inflammatory State Enriched in Pathobionts with Limited TLR Stimulation
Source: Diagnostics (Basel). 2020 Oct 28;10(11):879. doi: 10.3390/diagnostics10110879 (PMC7692151; doi:10.3390/diagnostics10110879)
Supplement: Supplementary file 1 [file diagnostics-10-00879-s001.zip › Supplementary files/Supplementary information_AV_020920 (1).docx]

**Appendix 1: additional figures and tables**

**ADDITIONAL TABLES**

**Table S1:** features for microscopic scoring of wet-mounts (1)

| **Lactobacillary grades** | | |
| --- | --- | --- |
| Grade I | predominantly lactobacillary morphotypes (1-2 µm rod-shaped bacteria), with very few coccoid bacteria present | |
| Grade II | diminished lactobacillary flora, mixed with other bacteria. We subdivided this group into slightly disturbed, fairly normal (IIa) and moderately disturbed, rather abnormal (IIb) lactobacillary flora. | |
| Grade III | numerous other bacteria, with no lactobacilli present | |
| **BV score** | | |
| Presence of *Gardnerella* or *Mobiluncus* morphotypes and/or clue cells, in combination with the absence of lactobacilli (lactobacillary grade IIb or III) | | |
| Full-blown BV | granular flora throughout the smear and/or >20% clue cells (score 2) | |
| Partial BV | patchy streaks of granular BV, found adjacent to areas of less dense normal or AV flora in the same smear (score 1) | |
| Absence of BV | no signs of BV (score 0) | |
| **AV score** | | |
| Lactobacillary grades are basis for composite score to which any of the four following variables are added: leucocytes, toxic leucocytes, parabasal cells and background flora. | | |
| Normal | No signs of AV; score 0-2 |  |
| Light AV | Score 3-4 |  |
| Moderate AV | Score 5-6 |  |
| Severe AV | Score 7-10 (maximum score). |  |
| Desquamative inflammatory vaginitis | Score 8-10, can be seen as most extreme form of AV |  |
| ***Candida*** |  |  |
| *Candida* is recognized as hyphae (psuedomycelium), blastospores (budding yeasts, single spores), or atypical (doubtful *Candida* structures) | | |

**Table S2**: Biological materials (DNA, cell lines and bacterial cultures) used in qPCR and HEK cell experiment

|  | **Grown in** | **Culture conditions** | **Concentration** | **Estimated by** |
| --- | --- | --- | --- | --- |
| DNA | Not applicable | Not applicable | 0.75-136.8 ng/µl | Qubit™ (Thermo Fisher Scienctific) |
| *Lactobacillus crispatus* LMG12005 | de Man, Rogosa and Sharpe (MRS) broth (BD) | 37°C, micro-aerobically | 5.6. 10^8^ CFU/ml (and dilutions) | Plating |
| *Streptococcus agalactiae* ATCC49447 | Brain-Heart infusion broth (BD) | 37°C, micro-aerobically | 2.4. 10^7^ CFU/ml (and dilutions) | Plating |
| *Escherichia coli* LMG2093 | Lysogeny broth (BD) | 37°C, micro-aerobically | 1.1. 10^7^ CFU/ml (and dilutions) | Plating |
| *Staphylococcus aureus* MI/1310/1938 (MSSA) | Mueller-Hinton broth (BD) | 37°C, micro-aerobically | 1.7. 10^7^ CFU/ml (and dilutions) | Plating for DNA extraction/ Optical density for growth inhibition test |
| *Lactobacillus fermentum* AMB-V01^*^ | MRS broth (BD) | 37°C, micro-aerobically | range | Optical densitity at 600nm |
| *Lactobacillus helveticus* AMB-V03^*^ | MRS broth (BD) | 37°C, micro-aerobically | range | Optical densitity at 600nm |
| *Lactobacillus gasseri* AMB-V10^*^ | MRS broth (BD) | 37°C, micro-aerobically | range | Optical densitity at 600nm |
| *Lactobacillus crispatus* AMB-V12^*^ | MRS broth (BD) | 37°C, micro-aerobically | range | Optical densitity at 600nm |
| *Lactobacillus johnsonii* AMB-V23^*^ | MRS broth (BD) | 37°C, micro-aerobically | range | Optical densitity at 600nm |
| *Lactobacillus reuteri* AMB-V38^**^ | MRS broth (BD) | 37°C, micro-aerobically | range | Optical densitity at 600nm |
| *Enterococcus faecium* LMG8147 | Brain-Heart infusion broth (BD) | 37°C, micro-aerobically | range | Optical densitity at 600nm |
| *Streptococcus salivarius* LMG 11489 | Brain-Heart infusion broth (BD) | 37°C, micro-aerobically | range | Optical densitity at 600nm |
| THP-1 monocytes | RMPI-1640 (Gibco), supplemented with 10% fetal bovine serum (Perbio Science) | 37°C, 5%CO_2_, 100% humidity | 1.10^6^ cells/ml | EVE™cell counter (VWR) |
| HEK-Blue™ hTLR4 cells and HEK-Blue™ hTLR2-TLR6 cells | DMEM (Gibco) containing 4.5g/l glucose (Gibco), 10% fetal bovine serum (Perbio Science), 100µg/ml Normocin™ (Invitrogen), 1x HEK-Blue™ Selection (Invitrogen) and 2mM glutamine (Gibco) | 37°C, 5%CO_2_, 100% humidity | 2.5.10^5^ cells/well | EVE™cell counter (VWR) |

**^*^** These strains were isolated from the samples of the cohort described in this paper 50µl of sample was plated onto an MRS solid base and grown at 37°C for 48-72h. Colonies were picked at random, grown in MRS broth, stored in 25%glycerol at -80°C, and identified with by 16S Sanger sequencing and confirmed by whole genome sequencing as previously described (2).

**^**^** This strain was isolated in our lab in a similar fashion from a sample from another study: Oerlemans *et al*. (in preparation)

**Table S3**: Primer sequences used for *16s rRNA* sequencing and qPCR

| **General bacterial primers used for barcoded PCR for MiSeq and qPCR** | | | |
| --- | --- | --- | --- |
| **Target gene/ region** | **Name** | **Sequence^(***)^** | **Source** |
| V4 region of *16s rRNA* gene | **V4.SA701** | CAA GCA GAA GAC GGC ATA CGA GAT **AAC TCT CG**A GTC AGT CAG CC*G GAC TAC HVG GGT WTC TAA T* | Kozich *et al.* (2013)(3) |
|  | **V4.SA501** | AAT GAT ACG GCG ACC ACC GAG ATC TAC AC**A TCG TAC G**TA TGG TAA TTG T*GT GCC AGC MGC CGC GGT AA* |  |
|  | **V1V2. SC701** | CAA GCA GAA GAC GGC ATA CGA GAT **ACC TAG TA**A GTC AGT CAG CC*G CTG CCT CCC GTA GGA GT* |  |
|  | **V1V2.SC501** | AAT GAT ACG GCG ACC ACC GAG ATC TAC AC**A AGC AGC A**TA TGG TAA TTC G*AG AGT TTG ATC MTG GCT CAG* |  |
| **Specific primers used for qPCR** | | | |
| **Target gene** | **Name** | **Sequence** | **Source** |
| *Lactobacillus 16s rRNA* | **Lacto_ Forward** | TGG AAA CAG RTG CTA ATA CCG | Byun *et al*. (2004)(4) |
|  | **Lacto_ Reverse** | GTC CAT TGT GGA AGA TTC CC |  |
| *Streptococcus 16s rRNA* | **STREP_P1** | GCTACACACGTGCTACAATGGTT | Rumyantseva et al (2016)(5) |
|  | **STREP_P2** | CAGCCTACAATCCGAACCGAGATT |  |
|  | **STREP_P3** | CAGCCTACAATCCGAACCGAGACT |  |
| *Staphylococcus 16s rRNA* | **STAPH_F** | GCTACACACGTGCTACAATGGACAA |  |
|  | **STAPH_R** | CGTATTCACCGTAGCATGCTGATCTA |  |
| *Enterobacteriaceae 16s rRNA* | **ENT_P1** | GCGGCCCCCTGGACGAAGA |  |
|  | **ENT_P2** | GCCTCAAGGGCACAACCTCCAA |  |
|  | **ENTERO_P3** | CGGTTCAAGACCACAACCTCTAA |  |
| *Cytochrome C1* (human reference gene) | **CYC_ Forward** | CATGTCCCAGATAGCCAAGGA | Moretti *et al.* (2019)(6) |
|  | **CYC_ Reverse** | CTTGTGCCGCTTTATGGTGTAG |  |

(***) These primers consist of an Illumina adapter, a barcode (indicated in bold), a pad, a linker and a *16s rRNA* specific part (indicated in italics). Only one barcode combination is shown here, the other used barcodes (the SA and SB set) can be found in Kozich *et al.* (2013)(3).

**SUPPLEMENTARY FIGURES**

**Figure S1. Classification of groups based on microscopy** (A)Classification of samples into aerobic vaginitis (AV), bacterial vaginosis (BV) or the reference group with a normal (NL) wet-mount microscopy image (400x magnification, phase contrast microscope), as detailed in the Materials and Methods. In the normal wet-mount microscopy (Lactobacillary grade I, no signs of AV or BV (7)) some superficial epithelial cells and *Lactobacillus* morphotypes (rod-shaped bacteria of a few micrometers) are visible. In the AV microcopy image parabasal cells (rounded cells smaller than superficial epithelial cells with a large nucleus) and toxic neutrophils (smaller cells with granules) and few bacteria are visible. In the BV microscopy image, clue cells (epithelial cells covered with a biofilm) and a large number of bacteria, including *Gardnerella* morphotypes, can be seen.

A B


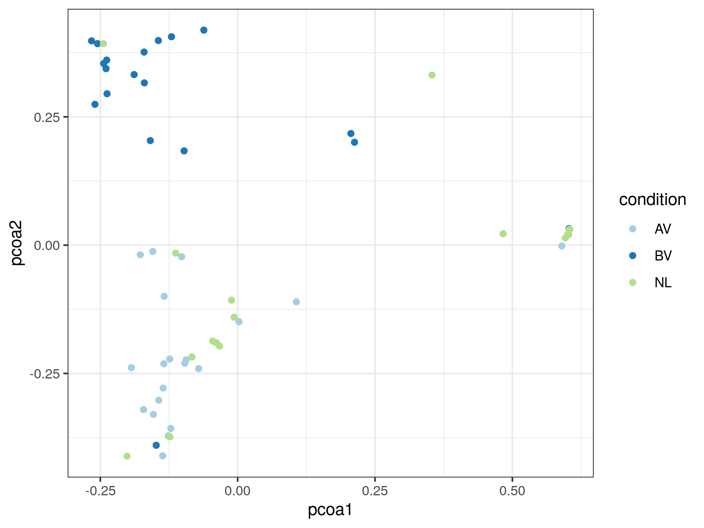

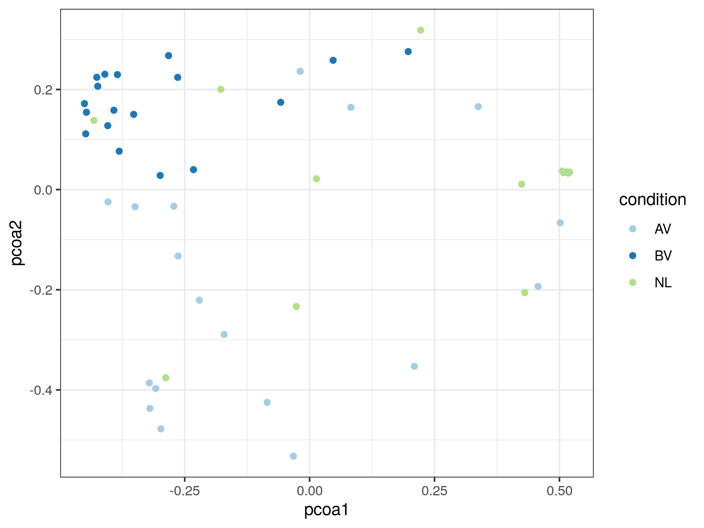


**Figure S2:** **PCOA plot indicating beta-diversity at ASV level (A) and genus level (B) between all samples of AV, BV and reference (NL) samples.** Beta-diversity was estimated by Bray-Curtis dissimilarity index. Samples are coloured by group.


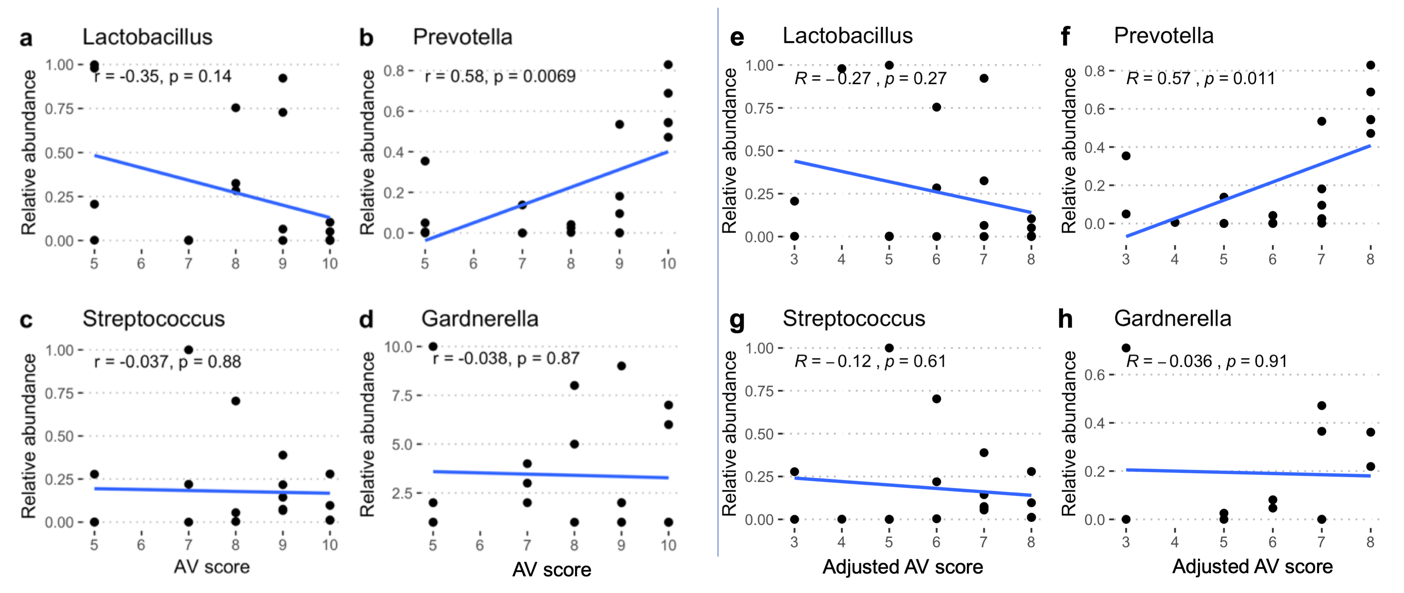


**Figure S3:** **Correlations between AV-scores and relative abundances bacterial taxa.** **(A-D)** Correlation between AV-score (A-D) or adjusted AV score (without Lactobacillary grade; E-H) and per sample cumulated relative abundances of *Lactobacillus* ASVs (A,E), *Prevotella* ASVs (B,F), *Streptococcus* ASVs (C,G), and *Gardnerella* ASVs (D,H). Pearson correlation coefficient and p-value are indicated.

A B


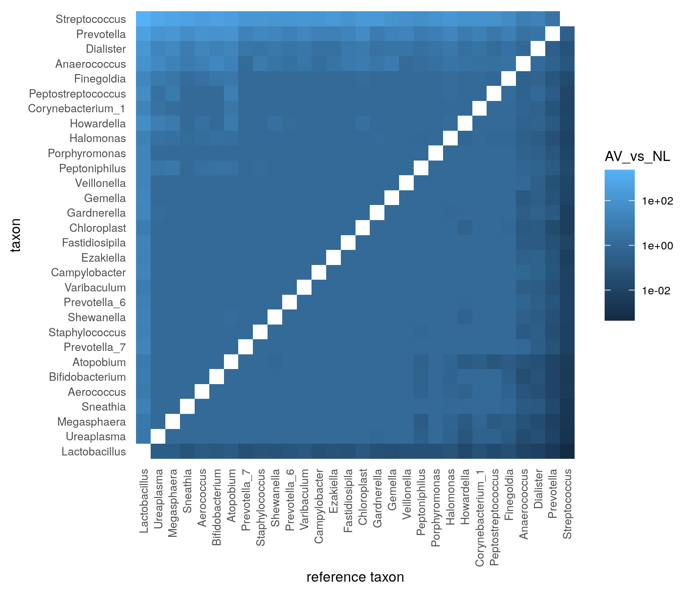

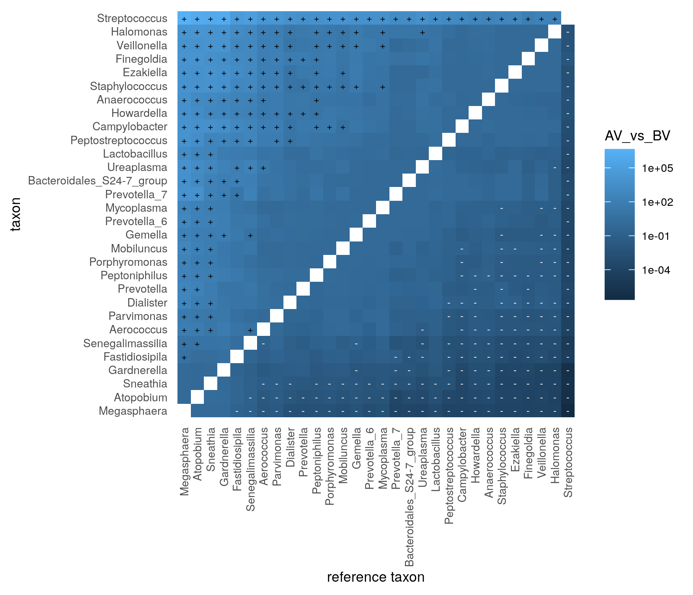


C D

**
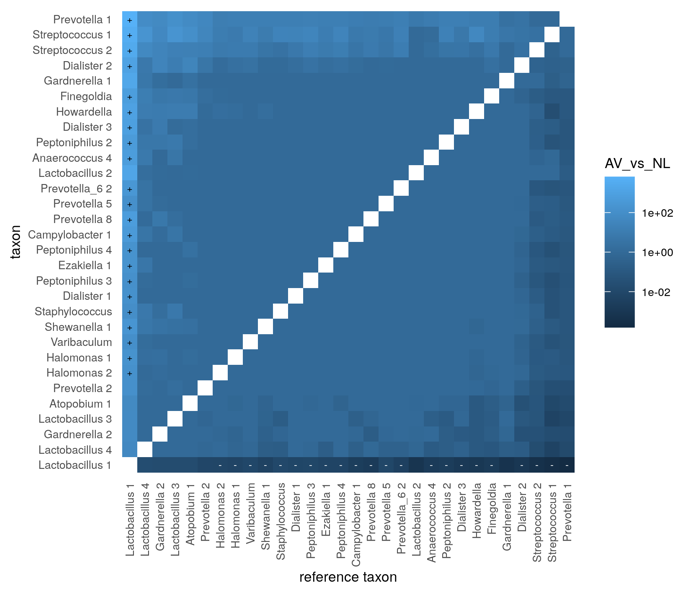

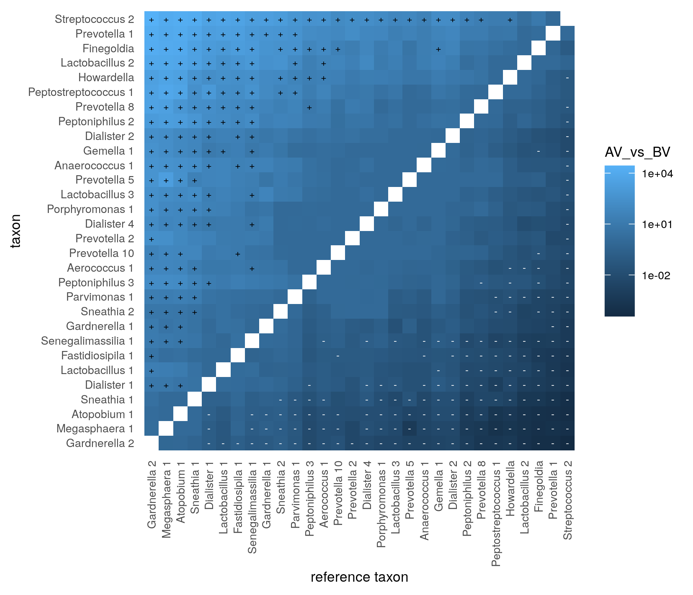
**

**Figure S4: Compositional analysis of differential abundance.** (A & C) Differential relative abundance for the most abundant genera of AV and NL samples at genus level (A) and ASV level (C) (B&D) Differential relative abundance for the most abundant genera of AV and BV samples at genus level (B) and ASV level (D). + or – indicate a significant difference for the specific genera.


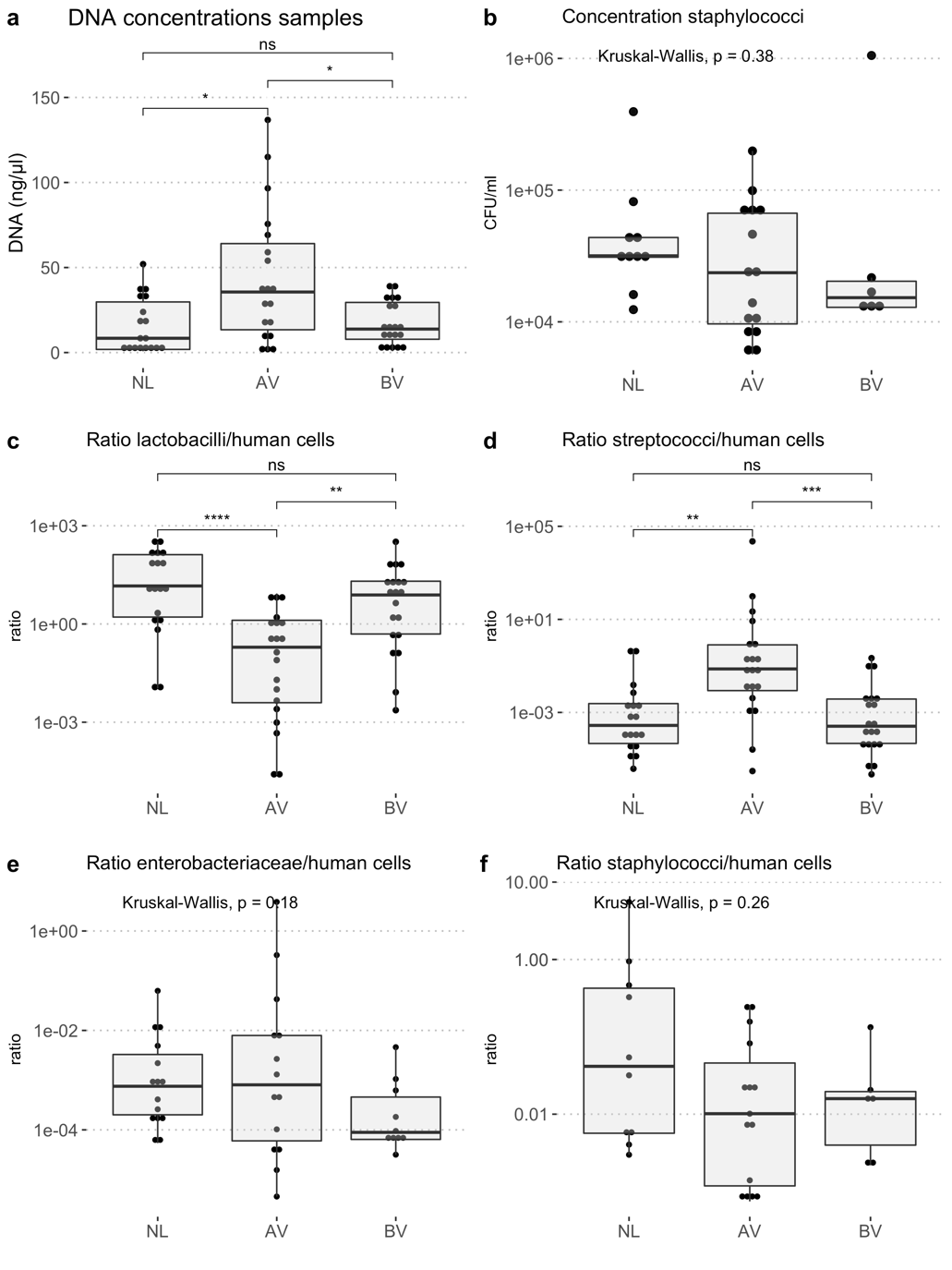


| ***p-values**** | ***DNA*** | | ***Staphylococci*** | | ***Lactobacilli/ human cells*** | | ***Streptococci/***  ***human cells*** | | ***Entero-bacteriaceae/ human cells*** | | ***Staphylococci/ human cells*** | |
| --- | --- | --- | --- | --- | --- | --- | --- | --- | --- | --- | --- | --- |
|  | **AV** | **BV** | **AV** | **BV** | **AV** | **BV** | **AV** | **BV** | **AV** | **BV** | **AV** | **BV** |
| BV | 0.024 |  | 0.91 |  | 0.002 |  | 0.0014 |  | 0.52 |  | 0.85 |  |
| NL | 0.022 | 0.484 | 0.57 | 0.54 | 0.0002 | 0.2637 | 0.0023 | 0.9654 | 1.00 | 0.12 | 0.43 | 0.44 |

**Figure S5:** **Supplementary information qPCR results.** **(A)** DNA concentrations of the samples, classified as reference (NL), AV and BV. (B) Estimated concentrations of staphylococci in the samples, based on qPCR results. Ratios of the concentrations of (C) lactobacilli to human cells, (D) streptococci, (E) *Enterobacteriaceae* and (F) staphylococci to human cells. To evaluate if there were significant differences in the groups, we performed Kruskal-Wallis and if significant this was followed by pairwise Wilcoxon tests to find which groups differed. In the graphs these significant differences based on the latter test were indicated unless no significant differences were obtained. In these graphs we indicated the p-value of the Kruskal-Wallis test. In the table the results of all the pairwise-Wilcoxon tests can also be found.

* based on pairwise Wilcoxon tests, adjusted by Holm’s method for multiple comparisons.


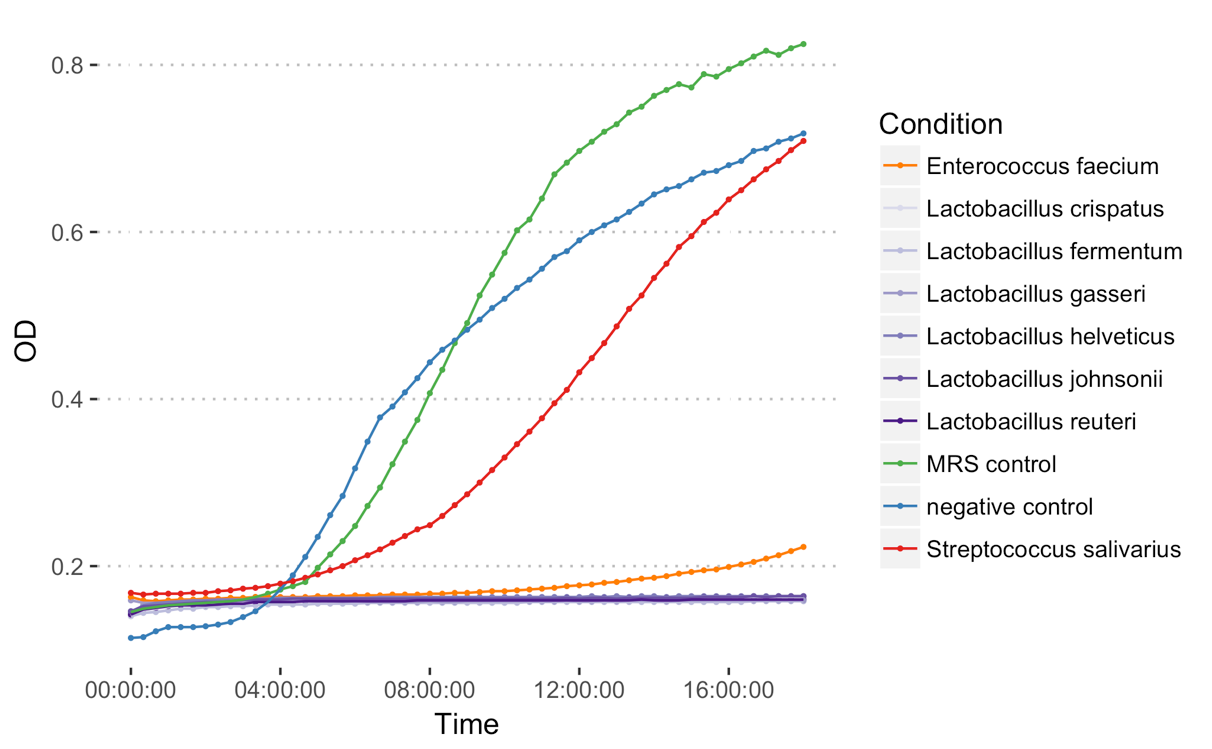


**Figure S6:** **Bacteriostatic effect of spent culture supernatant of vaginal lactobacilli on *S. aureus*.** Spent culture supernatant of overnight cultures of different vaginal *Lactobacillus* isolates, *Enterococcus faecium* and *Streptococcus salivarius* (Supplementary table S3) was obtained by centrifugation (2000g, 10 minutes) and passage of the supernatant through a sterile filter. These spent culture supernatants were added to the growth medium of *S.aureus* (Supplementary table S3) in a 1:5 ratio, which was subsequently inoculated with an overnight culture of *S.aureus* to a final concentration of 2%(v/v). As a negative control and MRS control, sterile distilled water and MRS was added to the growth medium, respectively, instead of spent culture supernatant. The growth of *S.aureus* was followed for 18h by measurements of the optical density at 600nm.

**REFERENCES**

1. Donders GGG, Vereecken A, Bosmans E (2002) Definition of a type of abnormal vaginal flora that is distinct from bacterial vaginosis : aerobic vaginitis. *BJOG An Int J Obstet Gynaecol* 109(1):34–43.

2. Wuyts S, et al. (2017) Large-Scale Phylogenomics of the *Lactobacillus casei* Group Highlights Taxonomic Inconsistencies and Reveals Novel Clade-Associated Features. *mSystems* 2(4):e00061-17.

3. Kozich JJ, Westcott SL, Baxter NT, Highlander SK, Schloss PD (2013) Development of a dual-index sequencing strategy and curation pipeline for analyzing amplicon sequence data on the miseq illumina sequencing platform. *Appl Environ Microbiol* 79(17):5112–5120.

4. Byun R, et al. (2004) Quantitative Analysis of Diverse Lactobacillus Species Present in Advanced Dental Caries. *J Clin Microbiol* 42(7):3128–3136.

5. Rumyantseva TA, Bellen G, Savochkina YA, Guschin AE, Donders GGG (2016) Diagnosis of aerobic vaginitis by quantitative real-time PCR. *Arch Gynecol Obstet* 294(1):109–114.

6. Moretti S, et al. (2019) Human inflammatory response of endotoxin affected by particulate matter-bound transition metals. *Environ Pollut* 244:118–126.

7. Donders GGG (2007) Definition and classification of abnormal vaginal flora. *Best Pract Res Clin Obstet Gynaecol* 21(3):355–373.
